# Direct Investigation of Covalently Bound Chlorine in Organic Compounds by Solid-State ^35^Cl NMR Spectroscopy and Exact Spectral Line-Shape Simulations''

**DOI:** 10.1002/anie.201200728

**Published:** 2012-03-14

**Authors:** Frédéric A Perras, David L Bryce

**Affiliations:** ' Department of Chemistry and CCRI, University of Ottawa10 Marie Curie Private, Ottawa, Ontario (Canada)http://mysite.science.uottawa.ca/dbryce/; '' F.A.P. thanks NSERC for a scholarship and D.L.B. thanks NSERC, CFI, and the Ontario MRI for funding. Access to the 900 MHz NMR spectrometer was provided by the National Ultrahigh-Field NMR Facility for Solidswww.nmr900.ca

**Keywords:** chlorine, DFT calculations, NMR spectroscopy, quadrupole effects, solid-state NMR spectroscopy

^35/37^Cl NMR spectroscopy studies of organic systems are very rare, with only a few neat liquids having been studied.^1^ The lack of chlorine NMR spectroscopy data may be explained by the fact that ^35^Cl and ^37^Cl are quadrupolar (spin *I*=3/2) and low-frequency isotopes. The quadrupole moments of the chlorine nuclei couple with the electric field gradient (EFG) tensor at the nuclei; this phenomenon is known as the quadrupolar interaction (QI). The quadrupolar coupling constant, *C*_Q_, and the quadrupolar asymmetry parameter, *η*_Q_, describe the magnitude and asymmetry of the QI. In solution, one of the consequences of the QI is fast relaxation, which means that the ^35/37^Cl NMR signals for covalently bound chlorines are very broad and are of low intensity.^1^ For these reasons, chemically distinct chlorine sites are very difficult to distinguish with solution NMR spectroscopy. However, in the solid state, nuclear spin relaxation is typically slower, thus enabling higher quality ^35^Cl NMR spectra to be collected, at least in principle. Unfortunately, the magnitude of the QI for covalently bound chlorines is very large because of the substantial, anisotropic EFG at the Cl atom, owing mainly to its electronic configuration when it forms a chlorine–carbon bond. Conventional wisdom is that such chlorine sites cannot be studied in powders by solid-state NMR spectroscopy as the central transition (CT; *m*_I_=1/2↔−1/2) can span tens of megahertz in typical commercially available magnetic fields. For this reason, only ionic chlorides^2^ and inorganic chlorides^3^ have been studied, as the EFG at these chlorides is often an order of magnitude smaller than at covalently bound chlorine atoms in organic molecules. The bonding environments for these types of chlorine atoms are substantially different from the environments in those chloride-containing molecules that have been studied previously.^2^, ^3^ A partial ^35^Cl NMR spectrum for hexachlorophene has been briefly mentioned in the literature.^4^ On the other hand, most of the interesting chlorine chemistry occurs when Cl is covalently bound to a carbon atom, where the chlorine atom often acts as a leaving group. Chlorine atoms are also important in many organic pharmaceuticals as well as in crystal design applications where they can form halogen bonds.^5^ Recent studies show that covalently bound chlorine is also important in biological chemistry where, for example, the tryptophan 7-halogenase was found to selectively chlorinate tryptophan moieties.^6^

Herein, we show that with the combination of an ultrahigh magnetic field (*B*_0_=21.1 T) and the state-of-the-art WURST-QCPMG pulse sequence,^7^ it is possible to acquire high-quality ^35^Cl NMR spectra of organic compounds that contain a covalently bound chlorine atom in powder samples in a reasonable amount of time. We have acquired ^35^Cl NMR spectra of 5-chlorouracil (**1**); the pesticide 2-chloroacetamide (**2**); sodium chloroacetate (**3**); α,α′-dichloro-*o*-xylene (**4**); chlorothiazide (**5**), a diuretic pharmaceutical also known as diuril; 2,4′-dichloroacetophenone (**6**); and *p*-chlorophenylalanine (**7**), a chlorinated amino acid, which is used as an inhibitor of tryptophan hydroxylase. These were chosen as a representative subset of compounds wherein chlorine is bound to sp^2^- or sp^3^-hybridized carbons. The molecular structures are shown in Figure 1 along with the NMR spectra. The ^35/37^Cl CT NMR spectra span on the order of 7 MHz at 21.1 T, necessitating the variable-offset cumulative spectral (VOCS) acquisition approach.^8^ Interpretation of the broad spectra of the CT requires line-shape simulations. Such line shapes are typically simulated by using second-order perturbation theory, where the QI is assumed to act as a perturbation to the Zeeman interaction. It is known from nuclear quadrupole resonance (NQR) studies that the values of *C*_Q_(^35^Cl) for covalently bound chlorine atoms are on the order of −70 MHz.^9^ As the Larmor frequency (*ν*_0_) for the nucleus ^35^Cl at 21.1 T (corresponding to a 900 MHz ^1^H *ν*_0_) is only 88.2 MHz, the ratio of *ν*_0_/*ν*_Q_ is around 2.5, where *ν*_Q_ represents the quadrupolar frequency. It is generally assumed that the high-field approximation is only valid if this ratio is higher than 10; thus, second-order perturbation theory is not expected to be valid to model the ^35^Cl NMR spectra shown herein, and an exact description needs to be used.^10^ Presently, we have written a new fast and graphical exact NMR simulation program, the technical details of which will be described elsewhere.

**Figure 1:**
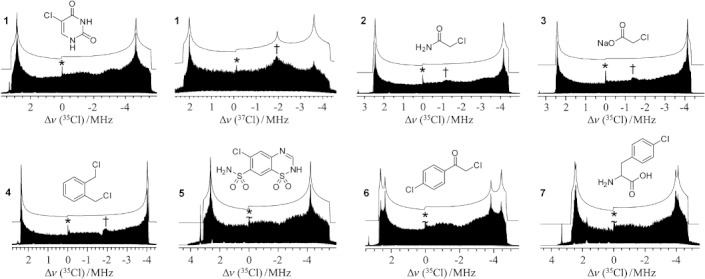
^35/37^Cl WURST-QCPMG NMR spectra (bottom traces), exact simulations (top traces), and chemical structures of compounds **1**–**7**. An asterisk is used to indicate a trace NaCl or NH_4_Cl impurity, whereas a cross marks a singularity from the satellite transition of the other chlorine isotope. The sharp lines on the high-frequency ends of the spectra are caused by radio interference.

The spectral simulations of the chlorine NMR spectroscopy data obtained with our QUEST (“QUadrupolar Exact SofTware”) program are shown in Figure 1. To demonstrate the critical importance of using exact Hamiltonian diagonalization for the interpretation of these NMR spectra, we have compared our simulations with those obtained using second-order perturbation theory. The high-frequency singularities are well-reproduced with the use of perturbation theory, but the low-frequency part of the spectrum appears stretched, which introduces an error in the chemical shift on the order of 600 ppm and an underestimation of the quadrupolar coupling constant, *C*_Q_, on the order of 700 kHz (Figure 2). These are of course non-trivial errors that would severely alter the interpretation of the NMR spectrum in terms of the chemical environment of the chlorine. Returning to Figure 1, it is interesting to note that the satellite transition (ST; *m*_I_= −3/2↔−1/2) of the ^37^Cl isotope overlaps with the observed CT of the ^35^Cl NMR spectrum. A singularity from this ST often appears in the ^35^Cl NMR spectra; in Figure 1 the singularities from the ST are marked with a cross. For compound **1**, which yielded the best signal-to-noise ratio per unit time of the samples studied, a ^37^Cl NMR spectrum was also acquired. This spectrum permitted us to perform an additional verification of the accuracy of the fits obtained with QUEST. As the ratios of the Larmor frequencies and quadrupole moments for ^35^Cl and ^37^Cl are known, the two NMR spectra can be related to one another. The ^37^Cl NMR spectrum of **1** is also shown in Figure 1. In this spectrum, a third singularity is present, which originates from the ST of the ^35^Cl isotope. As the STs are also strongly affected by third-order quadrupolar effects, this feature could not have been reproduced with the use of second-order perturbation theory.

**Figure 2:**
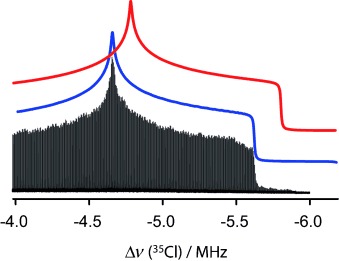
Low-frequency edge of the ^35^Cl NMR spectrum of **1**, showing a comparison between exact theory (blue) and second-order perturbation theory (red) simulations.

Four of the selected samples contain chlorine atoms covalently bound to sp^3^-hybridized CH_2_ carbon atoms, and four samples contain chlorine atoms that are bound to sp^2^ carbon atoms in aromatic rings. All chlorine atoms bound to CH_2_ carbon atoms have isotropic chemical shift (*δ*_iso_) values ranging from 150 to 200 ppm, whereas those bound to aromatic rings had *δ*_iso_ values on the order of 300 to 350 ppm (Table 1). Moreover, as has been explained using Townes–Dailey theory,^11^ the back donation of π-electron density from the chlorine atom into the π system of the aromatic rings creates an EFG different from zero that is perpendicular to the plane of the ring and that differs from the EFG in the plane of the ring. This difference leads to a deviation of the EFG from axial symmetry; this deviation is evidenced by the non-zero value of the quadrupolar asymmetry parameter (*η*_Q_, which ranges from 0 to 1 and takes a value of 0 in the case of axial symmetry). In the compounds we have studied, the asymmetry parameters for the chlorine atoms bound to CH_2_ carbon atoms remain nearly axially symmetric (*η*_Q_=0.008–0.032), whereas those for the chlorine atoms bound to aromatic rings are significantly larger (*η*_Q_=0.073–0.139). Figure 3 shows a clear separation of the two types of compounds studied herein on the basis of their *δ*_iso_ and *η*_Q_ values. It is evident that valuable chemical information that is not available from standard ^35^Cl NQR experiments can be obtained from solid-state ^35^Cl NMR spectroscopy.

**Figure 3:**
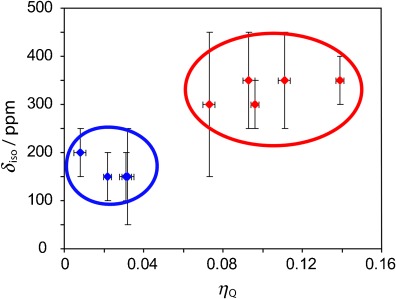
Scatter plot of the Cl chemical shifts and quadrupolar asymmetry parameters. The data points for chlorine atoms bound to CH_2_ groups are shown in blue, whereas those for chlorine atoms bound to aromatic rings are shown in red.

**Table 1:** ^35^Cl EFG tensor parameters and isotropic chemical shifts for covalently bound chlorine atoms.

Compound	*C*_Q_/MHz^[a,b]^	*η*_Q_	*δ*_iso_ [ppm]

**1**	−75.03±0.05	0.096±0.002	300±50
**2**	−68.30±0.05	0.031±0.003	150±50
**3**	−67.75±0.05	0.022±0.002	150±50
**4**	−66.43±0.08	0.008±0.003	200±50
**5**	−73.04±0.08	0.139±0.002	350±50
**6** (CH_2_)	−70.70±0.08	0.032±0.003	150±100
**6** (Ph)	−68.65±0.08	0.111±0.003	350±100
**7 a**^[c]^	−69.0±0.2	0.093±0.003	350±100
**7 b**^[c]^	−69.5±0.2	0.073±0.003	300±150

[a] Where *C*_Q_=*eQV*_33_/*h*, *η*_Q_=(*V*_11_-*V*_22_)/*V*_33_. Here, *e* is the fundamental charge, *Q* is the nuclear electric quadrupole moment, and *V*_11_, *V*_22_, and *V*_33_ are the principal components of the electric field gradient tensor.

[b] The absolute value of *C*_Q_ is obtained here, but it is known from theory and residual dipolar couplings that *C*_Q_ for terminal Cl atoms is negative. Chemical shift anisotropy was neglected.

[c] **7 a** and **7 b** refer to the two crystallographically distinct sites in compound **7**.

Interestingly, the breadth of the NMR line shapes enhances our ability to distinguish chemically distinct sites relative to solution NMR, as the powder pattern singularities are well separated. Compound **6** was chosen to test our ability to distinguish chemically distinct chlorine sites, because this compound has a chlorine atom directly bound to an aromatic ring (i.e., sp^2^) and another on a CH_2_ group (i.e., sp^3^). The singularities for both sites are well-separated and these can be simulated and assigned on the basis of their *δ*_iso_ and *η*_Q_ values. It also came as a surprise that two crystallographically distinct chlorine sites were observed for compound **7**, as two sets of horn singularities are present in the spectrum. The crystal structure of this compound is not known, although we can conclude, based on this NMR spectrum, that there are two non-equivalent molecules in the asymmetric unit. This conclusion is supported by our NQR studies as well (see below). This finding gives us an interesting perspective on the effect that crystal packing has on the NMR parameters. For the two chlorine sites, the value of *C*_Q_ varies by only 500 kHz (<1 % difference) and the value of *η*_Q_ differed by 0.02: these small differences are nevertheless manifested unambiguously in the ^35^Cl NMR spectrum of **7** despite its overall breadth.

It is interesting to compare this solid-state NMR spectroscopy method for probing the chlorine chemical environment with those that are already available. To that end, we have acquired ^35^Cl NQR spectra for all compounds (see Figure S1 in the Supporting Information). Unfortunately, only the quadrupolar product 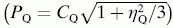 can be obtained by pure NQR methods with powder samples on nuclei with a spin of 3/2. Thus, precise values of *δ*_iso_, *C*_Q_, and *η*_Q_ cannot be obtained with that method. Importantly, as the respective ranges of *ν*_Q_ values for different chemical species (i.e., Cl atom bound to sp^2^ vs. sp^3^ carbons) effectively overlap, it is difficult, if not impossible, to obtain unambiguous chemical information from pure ^35^Cl NQR of these powdered samples.

Liquid-state ^35^Cl NMR spectroscopy on the other hand can directly provide only chemical shifts in favorable cases (e.g., neat liquids); however, site resolution is often lost owing to the breadth of the resonances relative to the chemical shift range of chlorine. With solid-state ^35^Cl NMR spectroscopy of powdered samples, we have shown that it is possible to capture the best of both methods while also gaining novel information about *η*_Q_, which appears to be the most distinctive NMR probe of the chemical environment of chlorine atoms.

Aside from acquiring complementary NQR spectra, we have also acquired ^13^C cross-polarization (CP) magic-angle spinning (MAS) NMR spectra for these samples (see the Supporting Information, Figures S2–S8). For five of the seven compounds we were able to resolve the residual dipolar coupled doublet associated with the carbon atom bound to a chlorine atom.^12^ The successful simulation of the doublets by using the values of *C*_Q_ from Table 1 provides further evidence for the validity of our exact Zeeman-quadrupolar diagonalization approach.

To our surprise, ^35^Cl WURST-QCPMG NMR spectroscopy at 21.1 T was extremely sensitive. A piece of an NMR spectrum could be acquired in a mere 8–20 min. In many cases, the acquisition of the full ^35^Cl NMR spectrum took less time than was necessary for the acquisition of the ^13^C CPMAS NMR spectrum at our standard field (9.4 T; see the Supporting Information). The relatively high sensitivity of this method shows that it may be feasible to look at larger systems where the chlorine concentration may be more dilute, e.g., in halogen-bonded systems, chlorinated peptides, and other chlorinated materials.

We have lastly investigated the observed trends with the use of gas-phase (B3LYP/6-311++G**)^13^ DFT calculations of the NMR parameters as well as solid-state, periodic, GIPAW DFT calculations^14^ (see Figure 4). Contrary to previous studies on other nuclei, the values of *η*_Q_ are much better reproduced with DFT than are the values of *C*_Q_. This difference is probably due to the fact that *C*_Q_ can vary by as much as 2 MHz depending on the temperature, whereas the *η*_Q_ value remains relatively constant, as it depends mainly on the symmetry along the bond.^15^ The chemical shifts are also very well reproduced by DFT, and are in support of the trend we observed (i.e., variation in the chlorine chemical shift may indicate if the adjacent carbon atom is sp^2^- or sp^3^-hybridized). The agreement is significantly greater for GIPAW DFT calculations, which shows that long-range crystal packing effects play a non-negligible role in determining the chlorine magnetic shielding constants.

**Figure 4:**
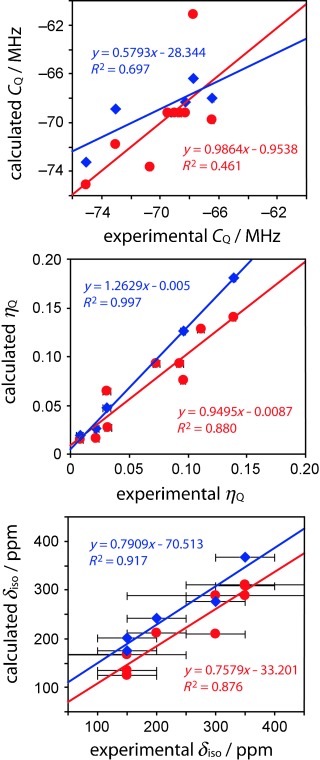
Correlation between calculated and experimental *C*_Q_, *η*_Q_, and *δ*_iso_ values. The red circles correspond to the results from gas-phase B3LYP calculations, whereas the blue diamonds correspond to the results from the solid phase GIPAW DFT calculations. Error bars for *C*_Q_ are within the size of the symbols.

In summary, we have shown that solid-state ^35^Cl NMR spectroscopy of purely covalently bound organic chlorine atoms can be used as a powerful and sensitive tool for structural investigations. The quadrupolar coupling constants are up to an order of magnitude larger than those reported for inorganic chlorides and organometallic chlorides, some of which exhibited partial covalent bonding character. The chemical shifts, and especially the quadrupolar asymmetry parameters, are very sensitive to structure, thereby making it possible to distinguish chemically different and even crystallographically different chlorine sites. To properly interpret the data, a program that describes the quadrupolar interaction exactly was necessary.

## Supporting Information

Detailed facts of importance to specialist readers are published as “Supporting Information”. Such documents are peer-reviewed, but not copy-edited or typeset. They are made available as submitted by the authors.
