# Supplementary material for: Direct Investigation of Covalently Bound Chlorine in Organic Compounds by Solid-State 35Cl NMR Spectroscopy and Exact Spectral Line-Shape Simulations''
Source: Angew Chem Int Ed Engl. 2012 Mar 14;51(17):4227–30. doi: 10.1002/anie.201200728 (PMC3430411; doi:10.1002/anie.201200728)
Supplement: Supplementary file 1 [file anie0051-4227-SD1.pdf]

Angewandte Chemie
Eine Zeitschrift der Gesellschaft Deutscher Chemiker

Supporting Information
© Wiley-VCH 2012
69451 Weinheim, Germany

Direct Investigation of Covalently Bound Chlorine in Organic Compounds by Solid-State ^{35}Cl NMR Spectroscopy and Exact Spectral Line-Shape Simulations**

*Frédéric A. Perras and David L. Bryce**

anie_201200728_sm_miscellaneous_information.pdf

Experimental

Chlorine-35/37 NMR experiments

All ^{35}Cl and ^{37}Cl NMR experiments were performed at 21.1 T using the Bruker AVANCE II spectrometer at the National Ultrahigh-Field NMR Facility for Solids in Ottawa. All samples were purchased from Sigma-Aldrich and used without further purification. For the chlorine NMR experiments, the samples were powdered and packed into 7 mm glass tubes which were then placed into the coil of a home-built 7 mm HX static probe. A WURST-QCPMG pulse sequence[1] was used with proton decoupling using 50 μs WURST pulses that swept over a frequency range of 1 MHz. The variable-offset cumulative spectral (VOCS) acquisition method was used,[2] where a series of 33 to 45 spectra were collected, processed and then co-added in the frequency domain. A QCPMG spikelet separation of 5 kHz was used in all cases with a VOCS stepping frequency of 200 kHz to ensure uniformly excited line shapes. Either 1000 (samples **1** and **2**), 1500 (samples **4** and **6**) or 2000 (samples **3**, **5**, and **7**) scans were used to acquire each piece using a recycle delay of 0.5 s. In most cases 128 echoes were collected for each QCPMG echo train (an acquisition time of 25.7 ms) although for **1**, **5**, and **6** it was possible to acquire 192 echoes (an acquisition time of 38.4 ms), thus increasing the signal intensity. The ^{37}Cl NMR spectrum of **1** was acquired using the same parameters as those used for ^{35}Cl although 1500 scans were collected. It was necessary to increase the attenuation of the pulses for a certain area in the spectrum (around -2.5 MHz) as the

[1] L. A. O'Dell, R. W. Schurko, *Chem. Phys. Lett.* **2008**, 464, 97-102.

[2] D. M. Massiot, I. Farnan, N. Gautier, D. Trumeau, A. Trokiner, J. P. Coutures, *Solid State Nucl. Magn. Reson.* **1995**, 4, 241-248.

power was not constant throughout the whole range. This varying power leads to "waves" in the lineshape around -1.5 and -3.5 MHz.

All the spectra were simulated using our new QUadrupolar Exact SofTware (QUEST) written in the C programming language which uses the fast matrix diagonalization function "gsl_eigen_hermv" from the Gnu Scientific Library (GSL)[3] as well as the space interpolation algorithm of Alderman, Solum, and Grant.[4] This enables the calculation of exact NMR/NQR spectra of quadrupolar nuclei in fewer than 2 seconds using a standard PC with a Pentium D processor with 2GB of RAM in a graphical user interface designed with QT C++. QUEST is a simulation program rather than a fitting program and the parameters need to be entered manually for every simulation. This program will be described in detail elsewhere. In all cases the effects of chemical shift anisotropy have been neglected. Their magnitude (of the order of 200 ppm according to calculations) is negligible when compared to the quadrupolar interaction and their effects on the line shape are non-observable.

Chlorine-35 NQR experiments

The ^{35}Cl NQR experiments were performed at 35°C using a 7 mm static NMR probe and an AVANCE III console. A Hahn-echo pulse sequence was used with a 3 μs excitation pulse length and a 6 μs refocusing pulse. In all cases the peak position was well predicted from the NMR data using the following equation.

---

[3] M. Galassi, J. Davies, J. Theiler, B. Gough, G. Jungman, P. Alken, M. Booth, F. Rossi, *GNU Scientific Library Reference Manual, Third Edition, for version 1.12*, Network Theory Ltd., United Kingdom, **2009**.
[4] D. W. Alderman, M. S. Solum, D. M. Grant, *J. Chem. Phys.* **1986**, 84, 3717-3725.

$$\nu_Q = \frac{C_Q}{2}\sqrt{1+\frac{\eta_Q^2}{3}}$$

An overlay of all the NQR spectra collected as a part of this study is shown in Figure S1.

Carbon-13 NMR experiments

^{13}C cross-polarization magic-angle-spinning (CPMAS) NMR experiments were performed at 9.4 T using a Bruker AVANCE III 400 spectrometer, although the ^{13}C CPMAS spectrum of chlorothiazide was provided to us by Dr. Victor Terskikh of the National Ultrahigh-Field NMR facility for Solids.[5] It was acquired using a Bruker DSX spectrometer and is described elsewhere.[5] All spectra were acquired using a 3.5 μs ^{1}H 90° excitation pulse and a 5 ms ramped amplitude contact time. A total of 44 to 500 scans were collected with recycle delays varying from 8 to 1200 s. The total acquisition times and signal to noise ratios are tabulated in Table S1 in comparison to the ^{35}Cl acquisition times and signal to noise ratios. In many cases, performing ^{35}Cl NMR is less time consuming than performing ^{13}C CPMAS NMR. The ^{13}C CPMAS NMR spectra for the 7 compounds in this study are shown in Figures S2-S8. For some of the spectra, it was possible to resolve the doublets caused by the residual dipolar coupling to $^{35/37}Cl$. These were then simulated using the known bond lengths and EFG tensor parameters.

[5] V. V. Terskikh, S. J. Lang, P. G. Gordon, G. D. Enright, J. A. Ripmeester, *Magn. Reson. Chem.* **2008**, 47, 398-406.

Computational

Cluster Model DFT Calculations

Cluster model (gas phase) calculations were performed at the B3LYP/6-311++G** level of theory using the Gaussian 09 program.[6] The clusters used included only a single molecule which was fully optimized using the same basis set and functional. The calculated EFG tensor parameters and chemical shifts are listed in Table S2. The calculated isotropic magnetic shielding values were converted to chemical shifts using the absolute shielding scale for chlorine determined by Wasylishen and co-workers ($\sigma_{iso}(Cl^-) = 974 \pm 4$ ppm).[7]

GIPAW DFT Calculations

Periodic DFT calculations were performed on compounds **1**-**5** as crystal structures were available.[8] These calculations used the gauge including projector augmented wave

---

[6] M. J. Frisch, G. W. Trucks, H. B. Schlegel, G. E. Scuseria, M. A. Robb, J. R. Cheeseman, J. A. Montgomery, Jr, T. Vreven, K. N. Kudin, J. C. Burant, J. M. Millam, S. S. Iyengar, J. Tomasi, V. Barone, B. Mennucci, M. Cossi, G. Scalmani, N. Rega, G. A. Petersson, H. Nakatsuji, M. Hada, M. Ehara, K. Toyota, R. Fukuda, J. Hasegawa, M. Ishida, T. Nakajima, Y. Honda, O. Kitao, H. Nakai, M. Klene, X. Li, J. E. Knox, H. P. Hratchian, J. B. Cross, C. Adamo, J. Jaramillo, R. Gomperts, R. E. Stratmann, O. Yazyev, A. J. Austin, R. Cammi, C. Pomelli, J. W. Ochterski, P. Y. Ayala, K. Morokuma, G. A. Voth, P. Salvador, J. J. Dannenberg, V. G. Zakrzewski, S. Dapprich, A. D. Daniels, M. C. Strain, O. Farkas, D. K. Malick, A. D. Rabuck, K. Raghavachari, J. B. Foresman, J. V. Ortiz, Q. Cui, A. G. Baboul, S. Clifford, J. Cioslowski, B. B. Stefanov, G. Liu, A. Liashenko, P. Piskorz, I. Komaromi, R. L. Martin, D. J. Fox, T. Keith, M. A. Al-Laham, C. Y. Peng, A. Nanayakkara, M. Challacombe, P. M. W. Gill, B. Johnson, W. Chen, M. W. Wong, C. Gonzalez and J. A. Pople, GAUSSIAN 03, (Revision C. 02), Gaussian, Inc., Wallingford, CT, **2004**.

[7] M. Gee, R. E. Wasylishen, A. Laaksonen, *J. Phys. Chem. A*, 1999, **103**, 10805-10812.

[8] S. A. Barnett, A. T. Hulme, N. Issa, T. C. Lewis, L. S. Price, D. A. Tocher and S. L. Price, *New J. Chem.*, 2008, **32**, 1761; B. Kalyanaraman, L. D. Kispert and J. L. Atwood, *J. Cryst. Mol. Struct.*, 1978, **8**, 175; L. Elizabé, B. M. Kariuki, K. D. M. Harris, M. Tremayne, M. Epple and J. M. Thomas, *J. Phys. Chem. B*, 1997, **101**, 8827; R. Basaran,

(GIPAW) methodology as implemented in the CASTEP program. The PBE functional was used in all cases along with ultrafine settings and on the fly generated ultrasoft pseudopotentials. The calculated EFG tensor parameters and chemical shifts are given in Table S2. The calculated magnetic shielding was also converted to chemical shifts in this case using the same absolute shielding scale denoted in the above section.

S.-Q. Dou and A. Weiss, *Ber. Bunsenges. Phys. Chem.*, 1992, **96**, 1688; A. Johnston, J. Bardin, B. F. Johnston, P. Fernandes, A. R. Kennedy, S. L. Price, A. J. Florence, *Cryst. Growth. Des.* **2011**, 11, 405-413.

Tables and Figures

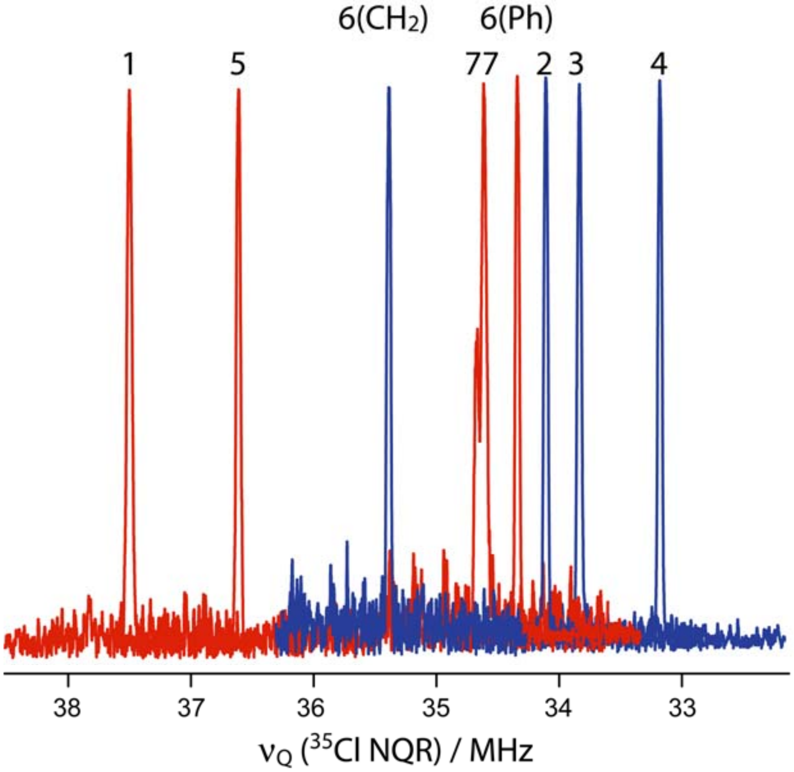

Figure S1. ^{35}Cl NQR spectra of the compounds **1**-**7**. The peaks are labeled in accord with their corresponding compounds. The blue spectra correspond to chlorine atoms bound to sp^3 carbons whereas the red spectra correspond to chlorine atoms bound to sp^2 carbons.

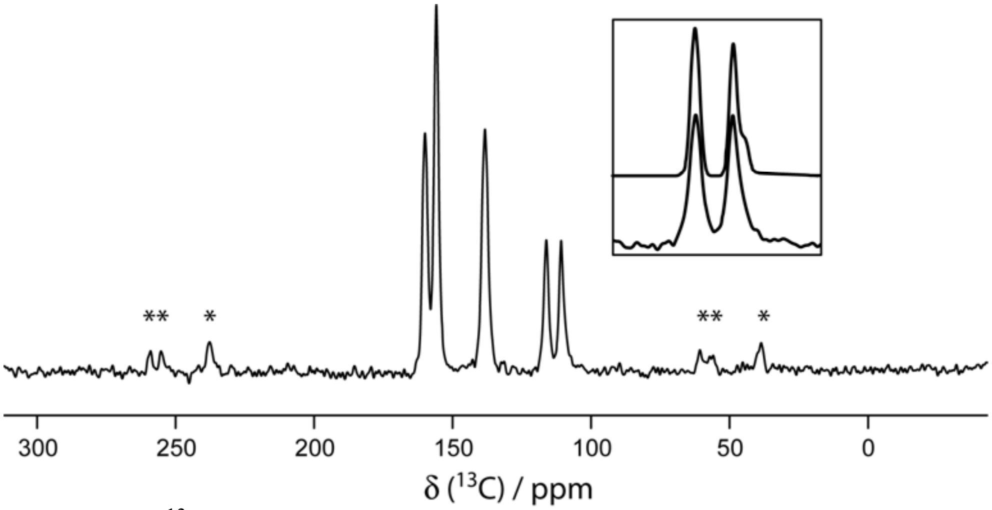

Figure S2. ^{13}C CPMAS NMR spectrum of compound **1**. An inset of the region around 120 ppm shows the doublet caused by the residual dipolar coupling to $^{35/37}$Cl and its simulation.

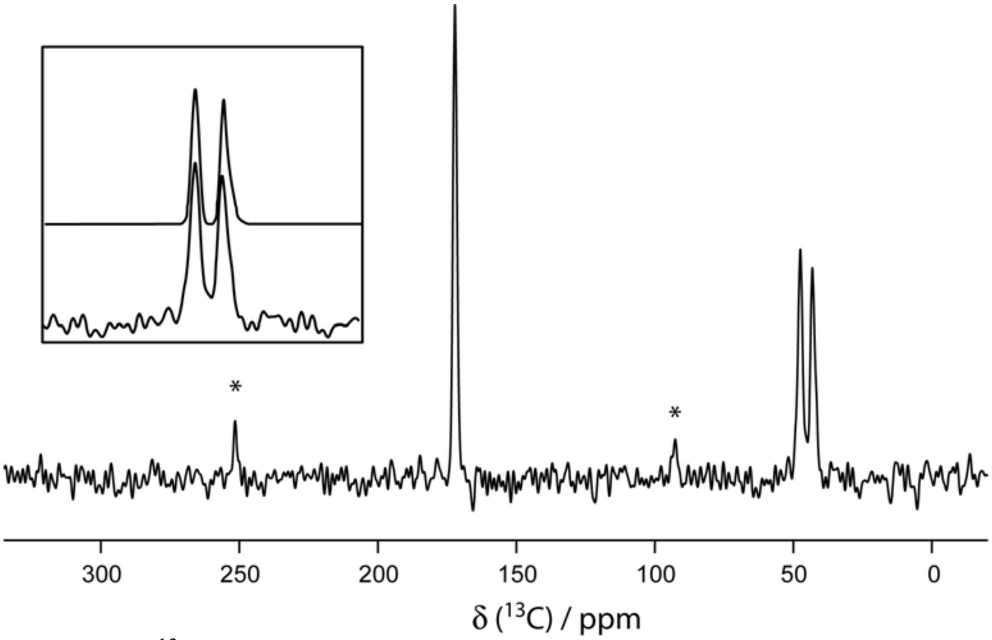

Figure S3. ^{13}C CPMAS NMR spectrum of compound **2**. An inset of the region around 45 ppm shows the doublet caused by the residual dipolar coupling to $^{35/37}$Cl and its simulation.

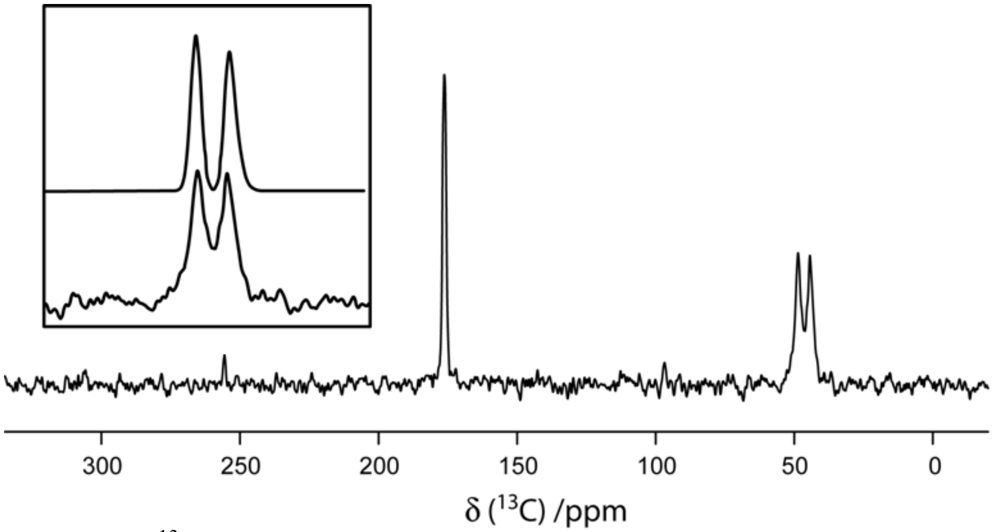

Figure S4. ^{13}C CPMAS NMR spectrum of compound **3**. An inset of the region around 45 ppm shows the doublet caused by the residual dipolar coupling to $^{35/37}Cl$ and its simulation.

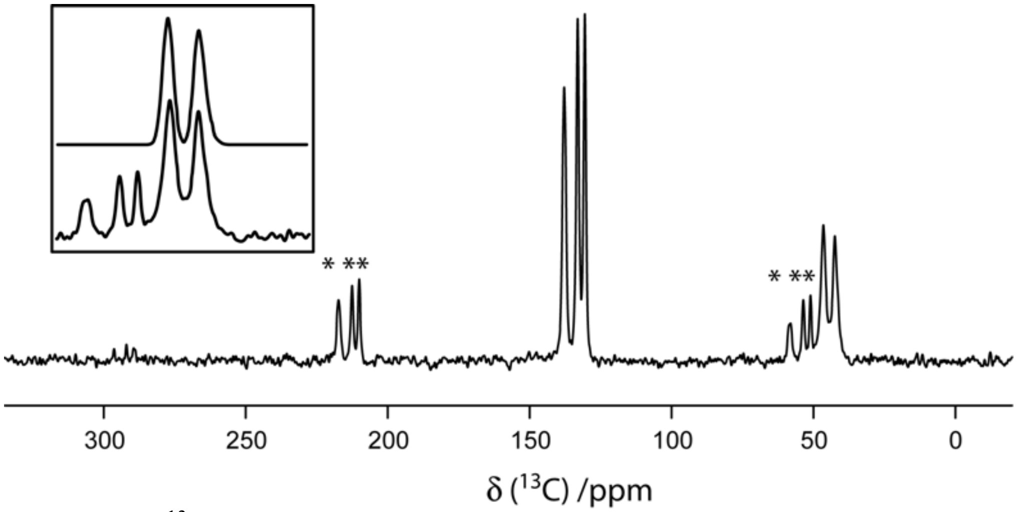

Figure S5. ^{13}C CPMAS NMR spectrum of compound **4**. An inset of the region around 45 ppm shows the doublet caused by the residual dipolar coupling to $^{35/37}Cl$ and its simulation.

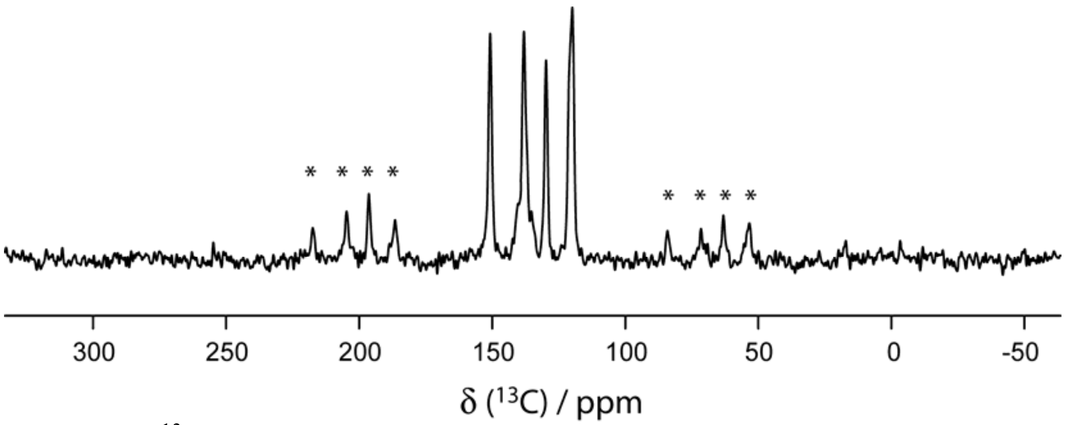

Figure S6. ^{13}C CPMAS NMR spectrum of compound **5**. In this case the residual dipolar coupled doublet is not resolved.

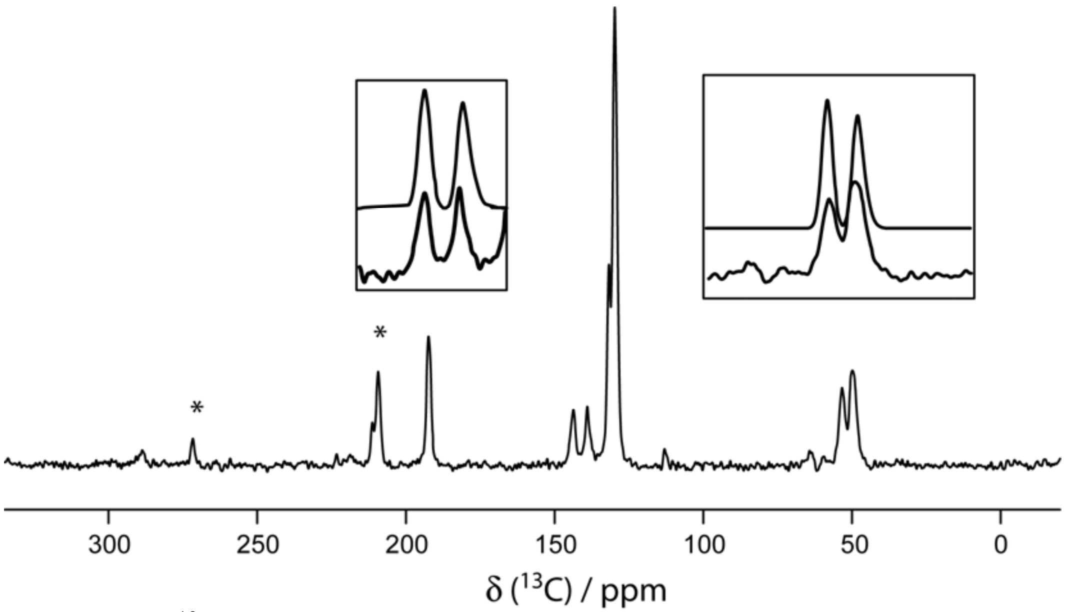

Figure S7. ^{13}C CPMAS NMR spectrum of compound **6**. Two insets of the regions around 140 and 50 ppm show the doublets caused by the residual dipolar coupling to $^{35/37}$Cl and their simulations.

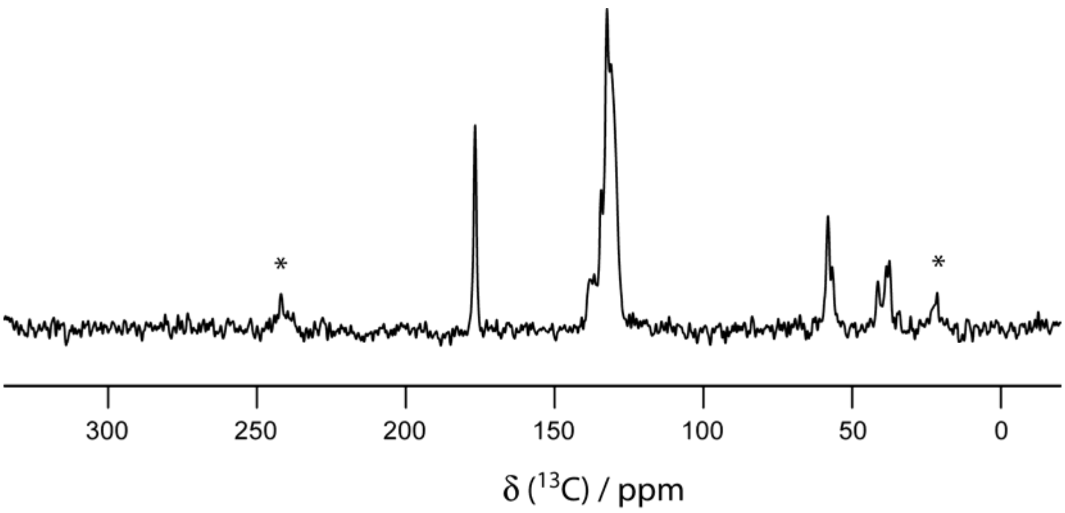

Figure S8. ^{13}C CPMAS NMR spectrum of compound **7**. The residual dipolar coupled doublet could not be resolved from the other peaks in this case.

Table S1. A comparison of the total experiment time necessary to acquire the ^{13}C CPMAS NMR spectra at 9.4 T and the ^{35}Cl WURST-QCPMG NMR spectra at 21.1T as well as the signal-to-noise ratios for both.

Compound	^{35}Cl NMR		^{13}C NMR	
	Total Exp. Time / h	Signal / Noise	Total Exp. Time / h	Signal / Noise
**1**	6.38	128.0	3.18	92.18
**2**	5.00	163.5	13.97	29.0
**3**	9.44	216.5	1.11	31.6
**4**	6.88	142.9	15.53	58.7
**5**	12.50	32.2	14.67	33.9
**6**	10.56	29.0	1.42	33.8
**7**	8.33	101.7	14.50	104.8

Table S2. Calculated EFG tensor parameters and chemical shifts for all the compounds studied.

Compound	C_Q / MHz		η_Q		δ_{iso} / ppm	
	Cluster model	GIPAW	Cluster model	GIPAW	Cluster model	GIPAW
**1**	-75.14	-73.24	0.076	0.126	209.5	277.1
**2**	-69.18	-68.28	0.065	0.047	135.1	174.5
**3**	-61.16	-66.35	0.016	0.026	124.5	201.6
**4**	-69.81	-67.97	0.015	0.019	211.5	241.8
**5**	-71.77	-68.92	0.140	0.181	308.1	367.0
**6 (CH_2)**	-73.69		0.027		167.2	
**6 (Ph)**	-69.21		0.128		311.4	
**7**	-69.23		0.093		287.4	
